# Evaluating the performance of commercial whole-genome marker sets for capturing common genetic variation

**DOI:** 10.1186/1471-2164-8-159

**Published:** 2007-06-11

**Authors:** Reedik Mägi, Arne Pfeufer, Mari Nelis, Alexandre Montpetit, Andres Metspalu, Maido Remm

**Affiliations:** 1 Institute of Molecular and Cell Biology, University of Tartu, Tartu, Estonia; 2 Institute of Human Genetics, Technical University Munich, Munich, Germany; 3 Estonian Biocentre, Tartu, Estonia; 4 McGill University and Genome Quebec Innovation Centre, Montreal, Canada; 5 The Estonian Genome Project Foundation, Tartu, Estonia

## Abstract

**Background:**

New technologies have enabled genome-wide association studies to be conducted with hundreds of thousands of genotyped SNPs. Several different first-generation genome-wide panels of SNPs have been commercialized. The total amount of common genetic variation is still unknown; however, the coverage of commercial panels can be evaluated against reference population samples genotyped by the International HapMap project. Less information is available about coverage in samples from other populations.

**Results:**

In this study we compare four commercial panels: the HumanHap 300 and HumanHap 550 Array Sets from the Illumina Infinium series and the Mapping 100 K and Mapping 500 K Array Sets from the Affymetrix GeneChip series. Tagging performance is compared among HapMap CEPH (CEU), Asian (JPT, CHB) and Yoruba (YRI) population samples. It is also evaluated in an Estonian population sample with more than 1000 individuals genotyped in two 500-kbp ENCODE regions of chromosome 2: ENr112 on 2p16.3 and ENr131 on 2p37.1.

**Conclusion:**

We found that in a non-reference Caucasian population, commercial SNP panels provide levels of coverage similar to those in the HapMap CEPH population sample. We present the proportions of universal and population-specific SNPs in all the commercial platforms studied.

## Background

Reduced genotyping costs and the availability of the International HapMap Project data [1] have made genome-wide association studies possible [2,3]. Multiple commercial SNP panels have been made available for large-scale studies. As the SNP selection strategies of these panels are different [4], it is important to know how well they can capture common variations in the human genome. Several studies have evaluated the "completeness" of these commercial panels on the HapMap population data [4-6]. The results of these studies indicate that most common SNPs are well captured, and despite substantial differences in marker selection strategies, the first-generation high-throughput platforms all offer similar levels of genome coverage [4,5].

The completeness with which variation is captured must also be evaluated for different populations. Unfortunately, the ethnicities of many patients sampled for complex disease gene identification projects will not be sufficiently reflected in the reference populations (CEU, YRI, CHB and JPT) selected by the International HapMap project. In addition, the number of genotyped individuals in HapMap populations is quite small, leading to under-representation of SNPs with lower allele frequencies. Some commercial panels have been designed using the limited data from HapMap. In this study, we have evaluated the performance of these commercial panels on HapMap populations and on one non-HapMap sample containing a large number of Estonian individuals. Estonia is a Northern European country that has been influenced by many waves of migration from Europe and Russia [7].

Several studies have already been performed to evaluate how well other Caucasian population samples can be described by tagSNPs calculated from HapMap CEPH data [7-10]. The authors of one study found that in three out of four selected gene regions, the tagSNPs of the CEPH population worked well on other European populations (> 70% markers had a r^2 ^≥ 0.8 with one of the CEPH tagSNPs) [8]. Another study found that 90–95% of Estonian SNPs with MAF > 5% have a r^2 ^of at least 0.8 with one of the CEPH tagSNPs [7]. In a third study, the authors suggest that CEPH samples provide an adequate basis for tagSNP selection in Finnish individuals [9]. The study by Gonzalez-Neira *et al*. [10] indicates that tagSNPs defined in Europeans are also efficient for describing Middle Eastern and Central/South Asian populations. Algorithms for tagging SNPs in multiple populations have been proposed by Howie *et al*. [11].

In view of this information, the aim of our study is to determine how well the recent commercial genome-wide genotyping arrays capture genetic variation in reference HapMap populations and in one non-HapMap population.

## Results

### The number of SNPs in the regions studied

One of our main aims was to compare the tagging performances of different commercial platforms on a non-HapMap population, specifically an Estonian population. As the Estonian individuals were genotyped only in two genomic regions we had to limit the analysis to these regions. The Estonian genotypes in our study originated from one gene-rich and one gene-poor ENCODE region (ENCODE regions of Chromosome 2: ENr112 on 2p16.3 and ENr131 on 2p37.1). In these regions, Yoruban, Asian and CEPH population samples contained 4540, 4495 and 4670 genotyped SNP assays, respectively (Table 1), in the final HapMap version 21. The number of genotyped SNPs in the Estonian population sample was 1420 (Table 2). These SNPs were randomly selected from the HapMap Phase I dataset. Among the CEPH, Asian and Estonian population samples the percentage of markers passing validation criteria was similar (49%, 48% and 54% for MAF ≥ 1%), but it was higher in the Yoruban population sample (68%), possibly because of the higher allelic diversity in African populations. Most of the SNPs that failed validation did so because of the low frequency of the minor allele (MAF < 1%).

**Table 1:** The number of SNPs used for calculations in each HapMap population sample

	YRI	CEU	ASI
All genotyped SNPs in regions	4540	4670	4495
			
Post validation markers:			
MAF = 1%			
minGeno ≥ 95%	3085	2293	2164
hwCutoff ≥ 0.001			
			
Post validation markers:			
MAF = 5%			
minGeno = 95%	2438	1912	1991
hwCutoff = 0.001			

The number of SNPs genotyped and the numbers of SNPs that passed validation criteria are shown. YRI, CEU and ASI (CHB + JPT) are, respectively, the Yoruban, CEPH and Asian (Chinese and Japanese) population samples from the HapMap data for two ENCODE regions of chromosome 2. MAF – minor allele frequency; minGeno – percentage of SNPs genotyped; hwCutoff – the p-value of the hypothesis that the current SNP is not in the Hardy-Weinberg Equilibrium.

**Table 2:** The reduced number of SNPs used for calculations shown in Figure 2

	YRI	CEU	ASI	EST
All genotyped SNPs in regions	1406	1407	1407	1420
				
Post validation markers:	1070	744	720	767
MAF ≥ 1%				
minGeno = 95%				
hwCutoff = 0.001				
				
Post validation markers:	835	627	661	605
MAF ≥ 5%				
minGeno = 95%				
hwCutoff = 0.001				

The number of SNPs genotyped and the numbers of SNPs that passed validation criteria are shown. YRI, CEU and ASI (CHB + JPT) are, respectively, the Yoruban, CEPH and Asian (Chinese and Japanese) population samples from the HapMap data for two ENCODE regions of chromosome 2. EST is the Estonian sample data from the same region. Counts for the YRI, CEU and ASI population samples show the counts of markers that were also genotyped in the Estonian population subset (1420 markers in total) and passed validation criteria. MAF – minor allele frequency; minGeno – percentage of SNPs genotyped; hwCutoff – the p-value of the hypothesis that the current SNP is not in the Hardy-Weinberg Equilibrium.

### Evaluating the performance of commercial marker sets in capturing the genetic variation of HapMap population samples

After selecting and validating SNPs, we compared the performances of commercial panels in two selected regions with those shown in other publications [4-6]. The comparison also gave us information about the performance of HumanHap 550 on HapMap populations that has not previously been published.

**Figure 1:**
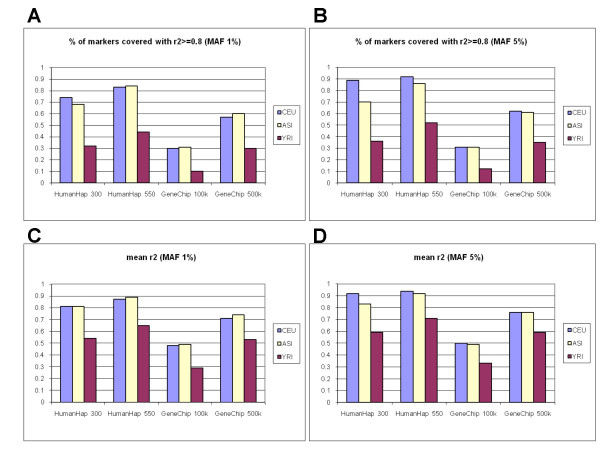
The performance of first-generation SNP panels on HapMap CEU, YRI and ASI population samples. The percentage of markers covered (A,B) and the mean r^2 ^between all SNPs and their best tagSNPs from each commercial panel (C,D) of CEPH, Yoruban and Asian HapMap population samples in two ENCODE regions (Chromosome 2: ENr112 on 2p16.3 and ENr131 on 2p37.1). To correct for the overestimate of coverage, we used the same correction as described by Barrett *et al*. 2006 [4].

To evaluate performance of commercial panels, for each marker present in HapMap data we calculated the best tagging SNP from each commercial panel. Then (a) the percentage of SNPs covered with r^2 ^≥ 0.8, and (b) the mean r^2 ^between each marker and their best tagging SNP for the investigated population was calculated. This was done for all population samples with two minor allele frequency cut-offs (1% and 5%). As shown in Figure 1 A–B, all commercial whole-genome SNP sets have poor coverage on the Yoruban population, whereas coverage of the CEPH and Asian populations can reach 80–90% on HumanHap 550. In addition to coverage in two ENCODE regions, the whole-genome coverage for commercial SNP panels was also evaluated as in the study by Barrett *et al*. 2006 [4]. The previously unpublished HumanHap 550 had the following whole-genome coverage estimations: CEU 86%, JTP + CHB 83%, YRI 48%. Among the technologies analyzed in this paper, HumanHap 550 had the best performance in all populations (Table 2). The advantage over HumanHap 300 is that HumanHap 550 has increased coverage in non-European populations. For other platforms, we observed coverage values nearly identical to previously published results (Table 3) despite some differences in data (HapMap ver.20 combined with Affymetrix genotypes on the HapMap samples vs. HapMap ver.21). The mean r^2 ^of the whole genome is shown on Table 3, the mean r^2 ^of two ENCODE regions is shown in Figure 1 C–D. In the Table 3, the r^2 ^value expresses the mean r^2 ^of all SNPs studied and additionally the r^2^of "covered" SNPs as in some previous studies [4]. Here again, HumanHap 550 shows higher values than other platforms, although the increase over HumanHap 300 is not large on the CEPH population.

**Table 3:** Genomic coverage, mean r^2^between tagged SNPs and their tagSNPs (calculated as in the study by Barrett *et al*. 2006 [4]) and mean r^2^of all SNPs and their tagSNPs. Common SNPs with MAF ≥ 0.05 were evaluated using Phase II HapMap (v. 21) data

	CEU			JPT + CHB			YRI		
	coverage (%)	mean r^2 ^tagged SNPs	all SNPs	coverage (%)	mean r^2 ^tagged SNPs	all SNPs	coverage (%)	SNPs	all SNPs
Illumina HumanHap 300	76	0.96	0.87	64	0.96	0.81	28	0.96	0.57
Illumina HumanHap 550	86	0.97	0.93	83	0.97	0.91	48	0.96	0.73
Affymetrix GeneChip 100 k	32	0.96	0.54	33	0.96	0.53	15	0.96	0.37
Affymetrix GeneChip 500 k	64	0.98	0.82	66	0.97	0.83	40	0.97	0.68

### Evaluating the performance of commercial marker sets in capturing the genetic variation in Estonian population samples

**Figure 2:**
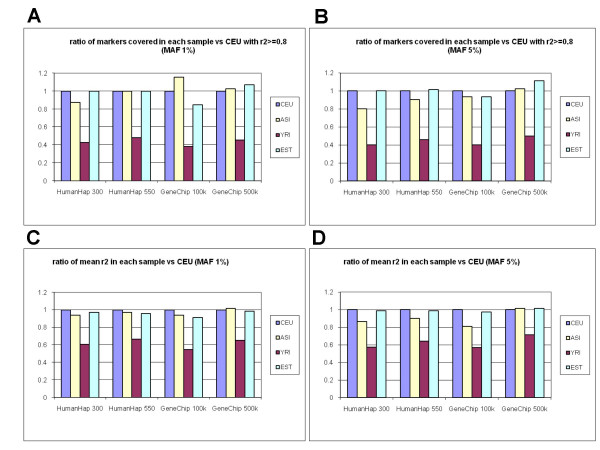
Evaluation of the performance of first-generation SNP panels in capturing common variance among the Yoruban, Asian and Estonian population samples compared to the CEPH population sample. The relative ratio of markers covered (A,B) and the relative ratio of mean r^2 ^between markers and their best tagSNPs (C,D) for the CEPH, Asian, Yoruban and Estonian population samples in two ENCODE regions (Chromosome 2: ENr112 on 2p16.3 and ENr131 on 2p37.1). Only markers present in the Estonian population sample were used to measure the percentage of markers covered and the mean r^2 ^between markers and their best tagSNPs. The results are expressed relative to the CEPH population sample.

Since fewer SNPs were genotyped in the Estonian sample than in the HapMap populations, the mean r^2 ^and coverage of the CEPH, Asian and Yoruban population samples could not be compared directly with the Estonian one. Many tagSNPs from the commercial panels were not genotyped in the Estonian sample so their pairwise LD could not be calculated for the Estonian markers. Our solution was to reduce the marker counts in the CEPH, Asian and Yoruban samples so that only the markers present in the Estonian dataset were used for pairwise LD calculation. By this means we could calculate the relative performances of the commercial platforms on the reduced SNP set (validated markers out of a total of 1420 genotyped in the Estonian population sample, see Table 2). The calculation was carried out for the CEPH, Asian, Yoruban and Estonian population samples and the results were expressed as fractions of the coverage of the CEPH sample (Figure 2A–D). The results show that the commercial products cover the SNPs investigated with the same efficiency in the Estonian, Asian and CEPH samples, but tagging performance was lower in the Yoruban sample.

### The fractions of universal and population-specific SNPs in commercial panels

**Figure 3:**
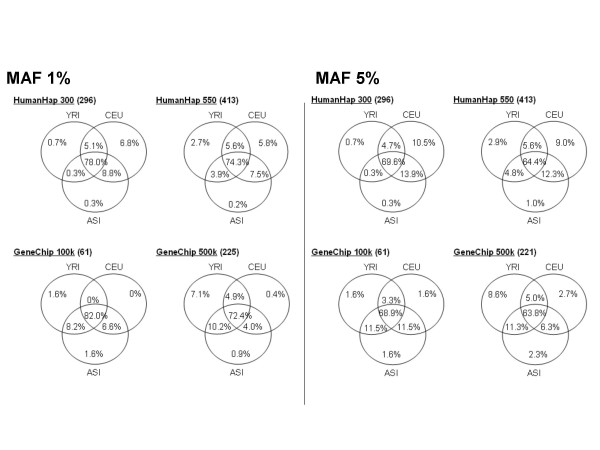
The number of commercial SNPs necessary to describe all SNPs in different populations. For each SNP in the commercial panels, we determined whether it was a tagSNP (the SNP with highest r^2^) for any marker in the selected population samples. For example, among the 296 SNP in HumanHap 300 with MAF 1% there were 231 (78%) SNPs that described SNP from all populations in these regions. Only 20 out of 296 (6.8%) were the best for describing the CEPH population and 2 (0.7%) were the best for describing only the Yoruban population. The analysis is based only on the two ENCODE regions in which the Estonian markers were genotyped.

It would be interesting to know how universal are the commercial panels for studying different populations. We counted the tagSNPs used for describing only one population and those that could identify SNPs from multiple populations (Figure 3 A–B). For each SNP in each population sample, the best-describing tagSNP from each of the commercial panels was identified. We then determined whether each commercial SNP was the best describer of all SNPs in one, two or all three populations.

Thus we were able to compare the universality of coverage of the different commercial platforms in different populations. We observed a strong bias towards CEPH-specific markers in the HumanHap 300 panel. This can easily be explained in terms of the SNP selection strategy used: markers were picked according to the CEPH HapMap population data using the r^2 ^based method [12], ensuring that the CEPH population has best coverage and thus contains more CEPH-specific SNPs. In contrast, GeneChip 100 K and GeneChip 500 K describe population-specific markers from all three populations fairly equally.

Our results show that universal markers constitute 63–82% of all SNPs and these numbers are similar in all the commercial platforms studied. Approximately 10% of the SNPs in commercial panels describe SNPs from only a single population sample.

## Discussion

In this study, two 500 kb ENCODE regions (0.3% of the genome) were used to find the efficiency with which a non-reference Caucasian population can be tagged by commercial SNP panels. As the whole-genome SNP coverage and the coverage of these two ENCODE regions are similar, we presume that these ENCODE regions are representative samples of the human genome. Estonian genotype data contain fewer commercial panel SNPs. Thus, several commercial panel SNPs were not genotyped and the LD between them and Estonian genotype data SNP could not be calculated. The lower density of commercial panel SNPs might reduce both coverage and mean r^2 ^values. To overcome the problem, similar HapMap reduced datasets were created and Estonian set was compared as a ratio vs. the CEU population results in Figure 2.

The results of our analysis show that the non-reference Caucasian population is tagged with the same efficiency as the CEPH population from HapMap. All non-African populations show similar levels of coverage in all commercial panels, irrespective of the SNP selection method for each platform. This is consistent with previous studies, which have shown that the CEPH population data from HapMap samples can successfully be used to tag other European population samples [7-10]. Other studies indicate that most of the common SNPs are captured by first-generation whole genome SNP panels [4,5]. Our study supports the combination of these results with another conclusion: commercial SNP panels can capture most of the common SNPs from non-reference European population samples. The new Illumina HumanHap 550 describes common markers slightly better than the smaller HumanHap 300 platform and reaches 86% coverage. Unfortunately, the remaining 14% of markers that are covered by r^2 ^< 0.8 can be quite numerous. If we assume that we would like to cover circa 7.5 million markers overall, 14% gives approximately one million poorly-covered markers. Any of these could be the disease-causing SNP that we are looking for in whole-genome association studies. Our hope is that upcoming commercial platforms will be able to cover most of these currently uncovered SNPs by additional tagSNPs.

In contrast to the results of previous studies [4,5], we observed equal or slightly smaller coverage in Asian and YRI population samples for Affymetrix 500 k than for Illumina HumanHap 300. However, this lower coverage may be due to the random variation of genomic regions; we used two 500 kb regions from the whole human genome. Some commercial panel SNPs can be used to tag markers from different populations. Other markers, however, are only useful for describing markers from a single population. The information about the universality of tagSNPs is important for planning association studies in non-HapMap populations. The markers that are able to tag different populations are expected to be useful in many populations. The fraction of universal markers (MAF > 1%) was found to be 72–82%.

## Conclusion

We found that in a non-reference Caucasian population, commercial SNP panels offered similar levels of coverage to the HapMap CEPH population sample. Although the coverage of commercial SNP panels has been evaluated for the HapMap CEPH population sample in previous papers, our results indicate that it is also possible to use that information for other European populations. We present the performance calculations for HumanHap 550, which have not previously been published. The coverage of HumanHap 550 reaches 90% of CEPH markers and 45% of Yoruban markers. We also present an analysis of the fraction of markers on commercial platforms that is universal and the fraction that is population-specific.

## Methods

### Data

Two previously resequenced 500-kb ENCODE regions on chromosome 2 (ENCODE 1: ENr112, NCBI Build 34 positions 51633239–52133238 on 2p16.3 and ENCODE 2: ENr131, NCBI Build 34 positions 234778639–235278638 on 2p37.1) were used in this study. These regions differ in their average recombination rates (0.8 cM/Mbp for ENCODE 1 and 2.1 cM/Mbp for ENCODE 2) and content of known genes (ENCODE 1 is a gene-poor region, whereas ENCODE 2 is a gene-rich region).

Overall, there are 2,431 and 2,067 SNPs in ENCODE 1 and ENCODE 2, respectively. These have been successfully genotyped in the HapMap project. From the two 500-kb ENCODE regions, 1420 SNPs were randomly selected and genotyped in 1090 samples from the Estonian Genome Project Foundation at McGill University and the Genome Quebec Innovation Centre, as part of the HapMap project, using the Illumina GoldenGate^® ^Assay. The total number of monomorphic SNPs was set at 100 for each region in all four HapMap populations included in the selection process. The same genotype data have previously been used in a study by Montpetit et al. [7].

For population comparisons, additional genotype data from CEPH (CEU, Utah residents with northern and western European ancestry), Asian (ASI, Mixed dataset of Japanese from the Tokyo area and Chinese from Beijing) and Yoruban (YRI, Yoruba people in Ibadan, Nigeria) populations of HapMap v. 21 were used, containing 4670 and 4540 SNPs respectively in these ENCODE regions.

### Marker validation

The markers for all three populations were validated using the Haploview program [13]. The population samples had to have genotyping success ≥ 95%, p-level of Hardy-Weinberg Equilibrium ≥ 0.001. Two minor allele cut-off levels were used (1% and 5%) to study the difference in results if markers with low allele frequency were present.

### TagSNP sets and evaluation of coverage

Information about the four evaluated commercial genome-wide genotyping arrays was retrieved from the manufacturers' websites: for the Infinium HumanHap 300 and HumanHap 550 Array Sets from Illumina, Inc [14], and for the Affymetrix GeneChip Mapping 100 K and the Mapping 500 K Array Sets from Affymetrix, Inc [15]. For analyzing the two ENCODE regions in HapMap populations (Figure 1 and 3) the following numbers of commercial panel SNPs were used: HumanHap 300, 296 SNPs; HumanHap 550,413 SNPs; GeneChip 100 k, 61 SNPs; GeneChip 500 k, 225 SNPs. For analyzing the Estonian dataset together with the reduced HapMap dataset (Figure 2) the following numbers of commercial panel SNPs were used: HumanHap 300, 118 SNPs; HumanHap 550,161 SNPs; GeneChip 100 k, 22 SNPs; GeneChip 500 k, 86 SNPs. Marker validation and LD calculations were performed using the Haploview [13] program.

Coverage numbers shown in Figure 1 and Table 3 were measured as a fraction of markers that had pairwise r^2 ^> = 0.8 with their best tagSNP from given commercial panel and its captured SNPs. To correct for the overestimate of coverage, we used the same correction as described by Barrett *et al*. 2006 [4].

To analyze how effectively the markers of different tag sets have been put to use, we determined the counts of tagSNPs used to describe each population and tagSNPs that could tag SNPs from multiple populations.

## Authors' contributions

RM performed the statistical analysis, created the figures and drafted the manuscript. AP initiated and helped to design the study, provided SNP data and was involved in drafting the manuscript. MN and AMo carried out the genotyping of the Estonian population samples under the supervision of AMe. MR participated in the design of the study and wrote the final version of the results and discussion. All authors read and approved the final manuscript.
